# Splenomegaly in Colon Cancer During Adjuvant Oxaliplatin-based Chemotherapy

**DOI:** 10.7759/cureus.7230

**Published:** 2020-03-10

**Authors:** Tulay Eren, Lale Pasaoglu

**Affiliations:** 1 Oncology, Diskapi Yildirim Beyazit Training and Research Hospital, Ankara, TUR; 2 Radiology, Ankara City Hospital, Ankara, TUR

**Keywords:** colon cancer, sinusoidal obstruction syndrome, splenomegaly, thrombocytopenia, oxaliplatin

## Abstract

Introduction

Oxaliplatin-based chemotherapy is the standard treatment in stage III colon cancer. Oxaliplatin may cause sinusoidal damage and sinusoidal obstruction syndrome (SOS) in the liver. Clinical reflections of SOS are splenomegaly and thrombocytopenia. This study aimed to investigate the frequency of splenomegaly development in patients receiving oxaliplatin-based chemotherapy and thrombocytopenia incidence rates related to this condition.

Materials and methods

Files of 50 patients having received fluorouracil and oxaliplatin (mFOLFOX6) regimen for stage 3 colon cancer between 2015 and 2017 were retrospectively reviewed. Spleen volumes (SV) of the patients were calculated using pre-and post-chemotherapy tomographic examinations. A 50% increase in the SV after chemotherapy (SV ≥1.5 change) was accepted as chemotherapy-associated splenomegaly. The patients were divided into two groups as having or not having splenomegaly after chemotherapy. Complete blood count was evaluated prior to each treatment cycle, and on the third month of treatment, termination was used for thrombocyte values.

Results

Splenomegaly was determined in 50% of the patients. Cumulative oxaliplatin dosage was found higher in those who developed splenomegaly (p = 0.003). Chemotherapy dose reduction was higher in patients who did not develop splenomegaly (p = 0.015). Thrombocytopenia was confirmed higher in patients who developed splenomegaly compared to those who did not (p = 0.047). Lower thrombocyte counts were found in the complete blood count of the patients having developed splenomegaly which was performed during and 3 months after chemotherapy when compared to those that did not develop splenomegaly (p-values, respectively, as 0.005, 0.038). Upon multivariate logistic regression analysis, cumulative oxaliplatin dose was the single independent factor related to splenomegaly (OR: 7.55 (1.90-31.61); p = 0.004).

Conclusion

Thrombocytopenia was confirmed to be higher in colon cancer patients receiving adjuvant mFOLFOX6 and developing splenomegaly during the post-treatment period. Moreover, splenomegaly was shown to be associated with the cumulative oxaliplatin doses received. Thrombocytopenia seen in patients receiving oxaliplatin for colon cancer should be a warning in terms of splenomegaly and SOS development.

## Introduction

Colon cancer is the third mostly encountered cancer in males and females and ranks second in cancer-related mortality [1]. The principle treatment method in stage 1-3 colon cancer is surgery. Postoperative adjuvant chemotherapy is the standard treatment method in stage III colon cancer [2]. Compared to 5-fluorouracil (5-FU)/leucovorin (LV) or capecitabine, oxaliplatin-containing regimens in stage 3 colon cancer adjuvant therapy have been shown to have contributing effects on survival [3].

Aside from the commonly known hematologic and nonhematologic side effects of oxaliplatin, it has rather significant side effects like peripheral neuropathy and hepatoxicity. The development of sinusoidal damage in the liver in patients receiving oxaliplatin was first confirmed in 2004 in patients receiving oxaliplatin-based chemotherapy prior to liver metastasectomy [4]. In the long term, patients receiving oxaliplatin may develop sinusoidal obstruction syndrome (SOS). The sinusoidal wall damaged by oxaliplatin toxicity causes congestive obstruction, which results in the destruction of sinusoidal blood flow [5-6]. It is known that SOS increases perioperative morbidity in patients having undergone liver resection, and thus, SOS detection is a crucial issue in patients to undergo liver resection [7].

Liver biopsy is the gold standard in confirming SOS diagnosis [8]. However, its clinical use is limited due to high cost, rare severe complication risk, and being an invasive procedure [8]. Studies have demonstrated that oxaliplatin-induced SOS causes an increase in the spleen dimension [9-10]. It has been established in colon cancer patients having undergone liver metastasectomy that hepatic sinusoidal damage risk prominently increases in those with a 50% or more enlargement of the spleen dimension following oxaliplatin-based chemotherapy [9]. Thrombocytopenia incidence in patients receiving oxaliplatin-containing chemotherapy regimens is 75% [11-12]. Though generally seen associated with bone marrow suppression, thrombocytopenia can also be rarely immune-associated [11,13]. One the other hand, Overman *et al*. have indicated that prolonged thrombocytopenia in patients receiving oxaliplatin may be associated with the changes oxaliplatin causes in the histologic structure of the liver and in the spleen dimension [9]. 

The aim of our study was to determine the rate of splenomegaly incidence and show the association between splenomegaly and thrombocytopenia in patients receiving oxaliplatin and 5-FU combination as adjuvant therapy due to colon cancer.

## Materials and methods

Patients

Data of 570 patients having received 5-fluorouracil, oxaliplatin, and calcium folinate (mFOLFOX6) in modified doses due to stage III colon cancer in the Ankara Numune Hospital Medical Oncology Clinic between 2015 and 2017 were retrospectively reviewed. Fifty patients had completed 12 cycles of mFOLFOX6 treatment, and patients having had control complete blood count on the 3rd month after chemotherapy and whose pre- and post-chemotherapy computed tomography (CT) data could be reached were included into the study. Exclusion criteria were as follows: not having completed adjuvant therapy, not having CT scans before and/or after chemotherapy, metastasis development during adjuvant therapy, having received additional chemotherapy agents to mFOLFOX6 treatment, having had a febrile diseases 3 months prior to adjuvant therapy, having another malignancy other than colon cancer, chronic liver disease, viral hepatitis B and C infections, alcohol-related liver disease, liver resection, or splenectomy history. 

Demographic, clinical, and laboratory data of the patients were obtained using a hospital automation system. Hematologic side effects of the patients were recorded examining the complete blood counts of the patients. In order to decrease the possibility of acute bone marrow suppression caused by chemotherapy, complete blood counts taken right before each chemotherapy application were used in detecting thrombocytopenia. 

Complete blood count tested on the 3rd month after chemotherapy was used to decrease the effect of chemotherapy-associated bone marrow suppression while evaluating long-term thrombocytopenia. Thrombocyte values were measured with Beckman Coulter LH-750 model complete blood count analyzer. Thrombocytopenia was described as thrombocyte count lower than 150x10³/µL.

Body mass index (BMI) was calculated as body mass (kg) divided by height (m^2^). BMI of 18.5-24.9 was accepted normal, 25-29.9 overweight, and 30 and over obese.

The performance status of the patients prior to chemotherapy was classified in accordance with the Eastern Cooperative Oncology Group (ECOG) performance status.

Necessary ethics board approval for the study was obtained from the Ethics Board of Ankara Numune Training and Research Hospital (Ethics board number: E-18-1898).

Chemotherapy protocol

Modified FOLFOX-6 consisted of oxaliplatin (85 mg/m^2^), LV (400 mg/m^2^), and a bolus of 5-FU (400 mg/m^2^) followed by a 46-hour infusion of 5-FU (2,400 mg/m^2^) repeated every 2 weeks. All patients received a total of 12 cycles. Cumulative oxaliplatin dosage was found by dividing total oxaliplatin dosages, given the entire chemotherapy cycles into body surface areas (BSA) of the patients. 

Calculation of spleen volume

Spleen volumes (SV) of the patients were measured by CT. Picture Archiving and Communicating Systems (PACS) were used to obtain CT images of the patients taken before and after chemotherapy. CT examinations were performed on a CT device with 16 slices (Aquilion 16, Toshiba Medical Systems, 2005, Japan). Parameters of the examination were: slice collimation 16 x 1.0 mm, rotation time 0.75 s, slice thickness 1 mm, 120 kV, and 300 mA. Nonionic 100 ml contrast matter (300 mg/ml) was administered with an automatic injector at 4 ml/s with an 18-20 G needle from the antecubital vein (Ulrich Medizin version, 2004, Germany). The imagings were evaluated by Vitrea® (4.1.52, 2014, Minnesota, USA) at the work station. SVs were calculated using semi-automatic organ volume calculation software at the work station before and after treatment separately. The marking on the organ volume calculation program was manually placed while calculating the SV. The software marked automatically using spleen density. The marked spaces that ran over the spleen were manually removed controlling in all images over the axial and coronal reformats of the spleen automatically colored by the software. The software automatically calculated SV as ml. This procedure was repeated for every patient in pre-and post-treatment images.

The radiology specialist was blind to the study. A 50% increase in the SV after chemotherapy (SV ≥1.5 change) was accepted as chemotherapy-associated splenomegaly. The patients were divided into two groups as having or not having splenomegaly after chemotherapy.

Statistical methods

The normality of the data was evaluated by the Kolmogorov-Smirnov test. Parametric data were expressed as mean ± standard deviation, non-parametric data as median (minimum-maximum), and categoric data as percentages. Mann-Whitney U test and Student’s t-test were used in the comparison of non-parametric and parametric data, respectively. Chi-square test or Fisher’s exact test was used in comparing categoric variables. Receiver-operating characteristics (ROC) analyses were used to determine the appropriate cut-off point for independent markers and calculate their sensitivity and specificity. Logistic regression analysis was performed to determine independent predictive factors for splenomegaly. IBM SPSS 20 (Statistics Program for Social Sciences) (USA) program was used for statistical analysis.

## Results

The mean age of the patients included in the study was 60.20 ± 8.46 years, and 64% of the patients were males. The patients were divided into two groups as those developing splenomegaly and those not. Splenomegaly was detected in 50% of the patients. The groups were similar in terms of age, sex, disease, performance status, and BMI distributions (Table 1). 

**Table 1:** Comparison of the demographic and clinicopathologic features of the patients BSA: body surface area; BMI: body mass index, ECOG: Eastern Cooperative Oncology Group

		Splenomegaly			p-value
		No (n = 25)	Yes (n = 25)	Total (n = 50)	
Age, Mean ± SD		62.08 ± 8.42	58.32 ± 8.24	60.20 ± 8.46	0.117
BSA (m²), median (min./max.)		1.70 (1.40/2)	1.70 (1.40/2)	1.70 (1.40/2)	0.742
BMI					
	Normal, n	7 (28)	8 (32)	15 (30)	0.877
	Overweight, n(%)	10 (40)	8 (32)	18 (36)	
	Obese, n(%)	8 (32)	9 (36)	17 (34)	
Sex					
	Male, n(%)	14 (56)	18 (72)	32 (64)	0.377
	Female, n(%)	11 (44)	7 (28)	18 (36)	
Tumor Grade					
	Good, n(%)	2 (8)	1 (4)	3 (6)	1
	Moderate, n(%)	21 (84)	22 (88)	43 (86)	
	Poor, n(%)	2 (8)	2 (8)	4 (8)	
Tumor localization					
	Right colon, n(%)	8 (32)	13 (52)	9 (18)	0.152
	Left colon, n(%)	17 (68)	12 (48)	12 (24)	
Pre-Chemo ECOG					
	0, n(%)	10 (40)	9 (36)	19 (38)	1
	1, n(%)	15 (60)	16 (64)	31 (62)	

Cumulative oxaliplatin dosage was detected higher in patients that developed splenomegaly (p = 0.003). Chemotherapy dosage reduction was higher in patients who did not develop splenomegaly (p = 0.015; Table 2).

**Table 2:** Cumulative oxaliplatin doses of the patients and dose reduction rates SD: standard deviation

		Splenomegaly			p-value
		No (n = 25)	Yes (n = 25)	Total (n = 50)	
Cumulative oxaliplatin dose (mg/m²), mean ± SD		906.88 ± 87.27	973.52 ± 72.02	940.20 ± 86.05	0.003
Dose reduction during chemotherapy					
	No, n(%)	4 (16)	12 (48)	16 (32)	0.015
	Yes, n(%)	21 (84)	13 (52)	34 (68)	

Thrombocytopenia was confirmed more in patients developing splenomegaly compared to those who did not develop splenomegaly according to thrombocyte values found in complete blood counts performed during chemotherapy (p = 0.047; Table 3).

**Table 3:** Thrombocytopenia incidence rates in patients that developed and did not develop splenomegaly

		Splenomegaly			P-value
		No (n = 25)	Yes (n = 25)	Total (n = 50)	
Thrombocytopenia					
	No, n(%)	9 (36)	3 (12)	12 (24)	0.047
	Yes, n(%)	16 (64)	22 (88)	38 (76)	
Thrombocytopenia at 3^rd^ month after chemotherapy					
	No, n(%)	15 (60)	13 (52)	28 (56)	0.569
	Yes, n(%)	10 (40)	12 (48)	22 (44)	

In control complete blood counts performed during and on the 3rd month after chemotherapy, thrombocyte count was detected lower in patients developing splenomegaly compared to those that did not (p-value, respectively, 0.005/0.038; Table 4).

**Table 4:** Comparison of thrombocyte counts in cases developing and not developing splenomegaly

Thrombocyte count (µL)	Splenomegaly		p-value
	No (n = 25)	Yes (n = 25)	
During chemotherapy, Median (Min/Max.)	136,000 µL (53,000/237000)	86,000 µL (57,000/152,000)	0.005
3^rd^ Month after chemotherapy, Median (Min/Max.)	160,000 µL (106,000/400000)	150,000 µL (67,000/224,000)	0.038

The optimum cut-off value of age and cumulative oxaliplatin dosage predictive of splenomegaly was determined by ROC analysis. The optimum cut-off value for age was 60 years, sensitivity 60% and specificity 56%; the same values for cumulative oxaliplatin dosage were, respectively, 94.4 mg/m^2^, 72%, and 68%.

In the comparisons carried out for those who developed splenomegaly and those who did not, multivariate logistic regression analysis was used to determine independent factors predicting splenomegaly and the parameters having a p-value of <0.20 (Table 5). As a result of the analysis, the only independent factor found in association with splenomegaly was the cumulative oxaliplatin dosage (OR: 7.55 [1.90-31.61]; p = 0.004).

**Table 5:** Multivariate logistic regression analysis of the factors affecting splenomegaly development CI: confidence interval, OR: odds ratio

		OR (%95 CI)	p
Age (years)			
	<60	1 (reference)	0.901
	≥60	1.09 (0.27-4.45)	
Localization			
	Right colon	1 (reference)	0.943
	Left colon	0.95 (0.19-4.48)	
Chemotherapy dose reduction			
	No	1 (reference)	0.137
	Yes	0.25 (0.04-1.56)	
Cumulative oxaliplatin dosage			
	<944 mg/m^2^	1 (reference)	0.004
	≥944 mg/m^2^	7.55 (1.90-31.61)	
Thrombocytopenia			
	No	1 (reference)	0.071
	Yes	4.65 (0.87-24.69)	
Thrombocytopenia at the 3^rd^ month after chemotherapy			
	No	1 (reference)	0.897
	Yes	1.10 (0.26-4.67)	

## Discussion

Splenomegaly development was determined in 50% of the patients having received adjuvant mFOLFOX6 chemotherapy for stage III colon cancer. Thrombocytopenia was encountered at a higher rate during the chemotherapy period in patients who developed splenomegaly (p = 0.047). Thrombocyte value measured during chemotherapy was significantly lower in patients that developed splenomegaly than those who did not (p = 0.005). Thrombocyte value measured in the 3rd month after chemotherapy was again significantly lower in patients who developed splenomegaly than those who did not (p = 0.038). Cumulative oxaliplatin dosage was found as the single independent factor that was in association with splenomegaly as a result of the multivariate logistic regression analysis conducted on predictive factors of splenomegaly (OR: 7.55 (1.90-31.61); p = 0.004).

Following the study by Rubbia-Brandt *et al*. who demonstrated the association between oxaliplatin use and SOS in metastatic colorectal cancer patients, an increase in oxaliplatin-associated spleen dimension has been started to be accepted as a common finding [4]. While splenic volume index (SVI) was used in the past to calculate SV after oxaliplatin-based chemotherapy, more accurate automatic measurements can be performed now on thin-sliced, high resonance CT [14-15]. Spleen dimensions can vary according to height, body surface area (BSA), and BMI [15]. In our study, there was no difference between the splenomegaly and non-splenomegaly groups in terms of age, BMI, BSA, and sex which are parameters that could affect spleen size.

There are different values in the literature regarding splenomegaly development rates during treatment in patients receiving oxaliplatin-based chemotherapy. Oxaliplatin-associated splenomegaly incidence in our study was found as 50%. Jung *et al*. have detected 67%, while Park *et al*. have detected 41.1% splenomegaly [12,16]. Splenomegaly is 24% in the study by Overman and 37.9% in the study by Kim *et al*. [9-10]. The reason is that the different rates of splenomegaly development might be due to the various techniques used in SV measurement. In our study, SV was measured using CT and software. In addition, features like race, height, weight, BSA, and BMI can be the causes of obtaining different results.

Studies have shown the relation between splenomegaly that develop in patients receiving oxaliplatin and thrombocytopenia [9-10,12]. Overman *et al*. have determined a strong correlation between splenomegaly development in patients receiving oxaliplatin and thrombocytopenia [9]. In the same study, the thrombocytopenic patient rate within the first year after chemotherapy has been found as 28% in patients who developed splenomegaly and 5% in those who did not develop splenomegaly, and these rates have been established as 13% and 0%, respectively, in the first year [9]. Moreover, it has been shown that patients who were thrombocytopenic at the end of year 1 were also thrombocytopenic at the end of year 3 [9]. Kim *et al*. have investigated the association between splenomegaly development in patients with colorectal cancer and thrombocytopenia receiving adjuvant FOLFOX therapy similar to ours. The authors have reported that thrombocyte count was lower both during the chemotherapy period and after in patients developing splenomegaly [10]. Moreover, it has also been shown that thrombocytopenia developing in splenomegaly patients was more severe than in those who did not develop splenomegaly [10]. Jung *et al*. have reported in colorectal cancer patients receiving FOLFOX4 a correlation between splenomegaly development and thrombocytopenia and emphasized that the correlation became more prominent after 6 cycles of chemotherapy [12]. Similarly, in our study, thrombocyte values were detected lower during and 3 months after chemotherapy in patients developing splenomegaly than in those who did not. The univariate analysis found a correlation between splenomegaly and thrombocytopenia, but this correlation was not observed on the multivariate analysis.

The studies have shown the association between splenomegaly development in patients receiving oxaliplatin and cumulative oxaliplatin dosage. In patients receiving oxaliplatin-based treatment, Park *et al*. have demonstrated a higher cumulative oxaliplatin dosage in patients with an increase in the spleen dimension compared to the patients without an increase in spleen dimension [16]. In another study, Overman *et al*. have obtained similar results and shown that the increase in the spleen dimension correlates with the cumulative oxaliplatin dosage [9]. Similarly, in our study, cumulative oxaliplatin dosage was detected higher in patients developing splenomegaly. In addition, cumulative oxaliplatin dosage was found as an independent factor for splenomegaly as a result of the logistic regression analysis. This result can be explained by the increase in sinusoidal damage occurring in the liver due to the high oxaliplatin dosage exposed. Splenomegaly was determined higher in patients who did not receive dose reduction during chemotherapy in our study, which can be explained by the increase in cumulative oxaliplatin dosage received by patients whose dosages were not reduced. Kim *et al*. have shown that splenomegaly developed more in patients receiving full-dose oxaliplatin than those receiving dose reductions, which is similar to our study [10].

There are some limitations to our study. We could only reach the pre-and post-chemotherapy tomography records of 50 patients who received adjuvant chemotherapy for stage III colon cancer. This is a reason for having a low number of patients. Since liver biopsy was not performed in our patients, splenomegaly which is an indirect indicator of SOS was chosen as the outcome. Since the peripheral smears of the patients were not evaluated, pseudotrombocytopenia could not be ruled out. In evaluating long-term thrombocytopenia, thrombocyte values checked in the 3rd month after chemotherapy were used and longer-term thrombocyte values were not reached in any patients.

## Conclusions

In conclusion, thrombocytopenia was detected to be seen at a higher rate in colon cancer patients receiving adjuvant mFOLFOX6 and developing splenomegaly after treatment. Moreover, splenomegaly was shown to be associated with the received cumulative oxaliplatin dosage. Thrombocytopenia seen in patients receiving oxaliplatin-based chemotherapy for colon cancer should be a warning in terms of splenomegaly and SOS development. 
