# Correction: Splenomegaly in Colon Cancer During Adjuvant Oxaliplatin-based Chemotherapy

**DOI:** 10.7759/cureus.c32

**Published:** 2020-06-17

**Authors:** Tulay Eren, Lale Pasaoglu

**Affiliations:** 1 Oncology, Diskapi Yildirim Beyazit Training and Research Hospital, Ankara, TUR; 2 Radiology, Ankara City Hospital, Ankara, TUR

A decimal point was erroneously included in the initial manuscript submission. This error was, regrettably, not caught during peer or editorial review. As a result, the decimal point has been removed from "94.4 mg/m2" as seen in the following sentence located in the results section: "The optimum cut-off value for age was 60 years, sensitivity 60% and specificity 56%; the same values for cumulative oxaliplatin dosage were, respectively, **94.4** mg/m2, 72%, and 68%."

The corrected sentence now reads: "The optimum cut-off value for age was 60 years, sensitivity 60% and specificity 56%; the same values for cumulative oxaliplatin dosage were, respectively, **944** mg/m2, 72%, and 68%."

Cureus sincerely regrets this error.

